# Prediction of out-of-pocket health expenditures in Rwanda using machine learning techniques

**DOI:** 10.11604/pamj.2020.37.357.27287

**Published:** 2020-12-21

**Authors:** Roger Muremyi, Dominique Haughton, Ignace Kabano, François Niragire

**Affiliations:** 1 African Centre of Excellence in Data Science, Department of Applied Statistics, University of Rwanda, Kigali, Rwanda,; 2 Global Studies, Bentley University, Boston, United States of America,; 3 Applied Statistics, University of Rwanda, Kigali, Rwanda

**Keywords:** MARS, out-of-pocket, Rwanda, validation, accuracy, training, testing

## Abstract

**Introduction:**

in Rwanda, the estimated out-of-pocket health expenditure has been increased from 24.46% in 2000 to 26% in 2015. Despite the existence of guideline in estimation of out-of-pocket health expenditures provided by WHO (2018), the estimation of out-of-pocket health expenditure still have difficulties in many countries including Rwanda.

**Methods:**

the purpose of this paper was to figure out the best model which predicts the out-of-pocket health expenditures in Rwanda during the process of considering various techniques of machine learning by using the Rwanda Integrated Living Conditions Surveys (EICV5) of 14580 households (2018).

**Results:**

our findings presented the model which predict the out-of-pocket health expenditures with higher accuracy and was found as treenet model. Furthermore, machine learning techniques were used to judge which predictor variable was important in our prediction process and comparison of the performance of the algorithms through train accuracy and test accuracy metric measures. Finally, the findings show that the tests of accuracy of the models were 50.16% for multivariate adaptive regression splines (MARS) model, 74% decision tree model, 87% for treenet model, 83% for random forest model, gradient boosting 81%, predictor total consumption played a significant role in the model for all tested models.

**Conclusion:**

finally, we conclude that the total consumption of the household came out to be the most important variable which is consistently true to all the algorithms tested. The findings from our study have policy implications for policy makers in Rwanda and in the world generally. We recommend the government to significantly increase public spending on health. Domestic financial resources are key to moving closer to universal health coverage (UHC) and should be increased on a long-term basis. In addition, these results will be useful for the future to assess the out-of-pocket health expenditures dataset.

## Introduction

The out-of-pocket health expenditures is one of the main issues in the policy decisions in developing countries. However, the rising health expenses in the emerging economies have drawn special attention toward building of suitable health shelter plans and basic healthcare facility that minimize the out-of-pocket health expenditures [1,2]. According to the World Health Organization, universal health coverage and access to affordable and good-quality health services is essential to human welfare and economic and social development [2]. Furthermore, health financing can be achieved through a variety of channels, including government budgets, donor funding, health insurance and direct payments.

In many countries´ direct payments, such as over-the-counter payments for medication, fees for doctors and services are the main forms of health financing, [3,4]. In fact, health problems are causing not only suffering and death but also negatively affect financial sides. Furthermore, the increase of out-of-pocket medical expenditures continues to be one of the world's biggest problems. In 2010, the World Health Organization estimated that, every year, 100 million people are pushed into poverty and 150 million suffer financial catastrophe because of out-of-pocket (OOP) expenditure on health services [4]. Furthermore, one of the high priorities in sustainable development goals (SDGs) was to provide universal health care services specifically for the populations of low- and middle-income countries [4,5]. In many low- and middle-income countries (LMICs), out-of-pocket health payments represent a significant portion of household expenditures. Consequently, the incidence of catastrophic out-of-pocket health payments, defined by the World Health Organization as exceeding 40% of household income, is linked to a violent cycle of impoverishment because households have to scale back spending on other necessities such as food and schooling [6].

In Rwanda, we observe a marked increase in out-of-pocket medical expenditures from 24.46% in 2000 to 26% in 2015 [7]. Despite, the policies that guide financial protection through community-based health insurance schemes (CBHIs) and other insurance providers, in Rwanda, the average amount to pay for health services at household level or at individual level is not well known [8]. Furthermore, some people are still suffering from an increase in out-of-pocket medical expenditures causing them delays in medical services and leading to permanent poverty and some households are facing challenges of paying medical bills or may delay getting healthcare services because of financial constraints [8,9]. Finally, one of barriers to accessing health services is the high cost of health care services [9]. One of the strategies adopted by the government of Rwanda that was based on health care reforms significantly contributed to the increase of health coverage; but there are still gaps in implementation and universal coverage has not yet been reached [9].

The purpose of this paper was to figure out the best model to predict the out-of-pocket health expenditures in Rwanda and results are geared to advance the field toward precise preventive care to lower overall out-of-pocket health expenditures and health access more efficiently. Moreover, for health insurers and increasingly healthcare delivery systems, accurate prediction of likely costs can help with general business planning [10]. Furthermore, for patients, knowing in advance their likely health expenditures for the following year could potentially allow them to choose insurance plans with appropriate deductibles and premiums [11]. There is limited evidence on the catastrophic and impoverishing effects of OOP health payments in Rwanda. Therefore, there is a need to provide evidence on the catastrophic and impoverishing effects of OOP health payments in Rwanda in order to inform governments and policy makers on the necessity of designing programs and policies that would provide financial risk protection to populations as a target of sustainable development goals (SDGs) [12]. Based on the above facts, a predictive analysis of the current out-of-pocket health expenditure with high accuracy is useful along the planning of the future and reduces the existing increase in health care cost.

Many studies have been conducted on predicting the out-of-pocket medical expenses using machine learning techniques in the world, but with none on the prediction of the out-of-pocket medical expenditures using machine learning techniques in Rwanda. Ortiz I *et al*. and Kawabata K *et al*. [13,14] estimates the out-of-pocket health expenditures in Rwanda by using two-part model to estimate the correlates of out-of-pocket expenditures for outpatient and inpatient care. However, a limitation of their findings includes the presence of biasedness caused by sample selection with respect to unobservable characteristics and the presence zero cost expenditures for health on some households. Moreover, they did not take into consideration of nonlinear relationship and interaction between variables. However, these issues will be solved and fixed using machine learning algorithms.

Kawabata K *et al*. [14] and Kronick R *et al*. [15], used gradient boosting and artificial neural network (ANN), for predicting healthcare costs on systematic literature review and empirical evaluation in the USA, their results found that gradient boosting had the best predictive performance overall and for low to medium cost individuals and for high cost individuals [16,17]. Moreover, artificial neural network stated that predicting healthcare costs for individuals using accurate prediction models were important for various stakeholders beyond health insurers and for various purpose [18]. Furthermore, for health insurers and increasingly healthcare delivery systems, accurate forecasts of likely costs can help with general business planning in addition to prioritizing the allocation of scarce care management resources. Finally, for patients, knowing in advance their likely expenditures for the next year could potentially allow them to choose insurance plans with appropriate deductibles and premiums [18,19].

The development of accurate healthcare cost prediction models using machine learning methods has been more recent [18]. According to Thomson S *et al*. [19] in their findings, classification tree and regression trees provide better predictions of healthcare costs compared to traditional statistics. Moreover, Thomson S *et al*. investigate regression trees to predict whether a household is going to incur higher or lower health care expenditure. The few studies conducted in Rwanda on out-of-pocket health expenditures faced some limitation, thus machine learning techniques have been used in this paper to improve the accuracy of estimates and prediction of out-of-pocket health expenditures in Rwanda.

**Health insurance out-of-pocket maximums:** in the health insurance industry, out-of-pocket expenses refer to the portion of the bill that the insurance company doesn't cover and that the individual must pay on their own [20].

**Machine learning:** machine learning (ML) is the study of computer algorithms that improve automatically through experience [21].

**Treenet or stochastic gradient boosting:** a treenet model normally consists of from several dozen to several hundred small trees, each typically no larger than two to eight terminal nodes. In addition, the model is similar in spirit to a long series expansion a sum of factors that becomes progressively more accurate as the expansion continues and improves decision tree models by summing a series of simple tree models,

$$F(X)=F_{0}+\sum_{i=0}^{M}\beta_{i}T_{i}(X)$$

where each T_i_ is a small tree [22].

**Multivariate adaptive regression splines (MARS):** the MARS model is essentially a linear statistical model with a forward stepwise algorithm to select model terms followed by a backward procedure to prune the model. The approximation bends to model curvature at knot locations and one of the objectives of the forward stepwise algorithm is to select appropriate knots and uses linear models with piecewise linear transformations of predictors to capture non-linear dependencies [22].

**Decision tree:** a decision tree is a tree like structure in which internal node represents test on an attribute, each branch represents outcome of test and each leaf node represent class label. Moreover, decision tree is a type of supervised learning algorithm (having a predefined target variable) that is mostly used in classification problems. Furthermore, it works for both categorical and continuous input and output variables [23].

**Out-of-pocket expenditure:** an out-of-pocket expense (or out-of-pocket cost) is the direct payment of money that may or may not be later reimbursed from a third-party source. Moreover, in the health care financing sector, this represents the share of the expenses that the insured party must pay directly to the health care provider, without a third-party [23].

**Random forest:** according to Wang W *et al*. [24] a random forest is a classification and regression model based on a forest of decision trees using random input to further improve predictive performance. However, random forest uses a forest of decision trees by selecting random subsets of predictors for each tree and bootstrap samples of the training set and is a supervised learning technique and as the name suggests, it forms forest-like structures with decision trees that are generated using the random sampling with replacement [24].

**Supervised learning algorithms:** supervised learning algorithms help the learning models to be trained efficiently, so that they can provide high classification accuracy. Moreover, the supervised learning algorithms support the search for optimal values for the model parameters by using large data sets without over-fitting the model. Therefore, a careful design of the learning algorithms with systematic approaches is essential. The machine learning field suggests three phases for the design of a supervised learning algorithm: training phase, validation phase and testing phase. Hence, it recommends three divisions of the data sets to carry out these tasks [24].

**Training:** training algorithms mainly help tune the model parameters and optimize them with labeled data sets. Training algorithms require quantitative measures to successfully train learning models using labeled data sets [23,24].

**Testing:** the testing of models is a process of evaluating the performance of the model trained by the training algorithm. In simple terms, we can say that the testing will confirm that the trained model works on a different data set (test data set), which is also labeled [24].

**Validation:** in general, the classification problem may be interpreted as the classification of seen data and the classification of unseen data. However, the best model should perform efficiently in both situations and the validation of models can help achieve the process. Moreover, the validation of a model may be defined as the testing of the model on multiple combinations of training and test data sets and aggregating the results. Now the challenge is how to generate or obtain multiple combinations of training and test data sets that can guarantee that the selected model will perform well with future data. The processes of generating such combinations of data sets and the statistical characteristics of the data determine the effectiveness of the validation algorithm [23,24].

**Root mean square error:** root mean square error is the average deviation of the predictions from the observations. Moreover, it is useful to get a gross idea of how well or not an algorithm is doing, in the units of the output variables [24]. According to Wang W *et al*. [24] in machine learning when we want to look at the accuracy of our model, we take the root mean square of the error that has occurred between the test values and the predicted values. However, let

$$a={\left(predicted_{i}-actual_{i}\right)}^{2}$$

and b=mean of a for a single value, then RMSE [24] is the square root of b which given by:

$$RMSE=\sqrt{\frac{\sum_{i=0}^{N}{\left(predicted_{i}-actual_{i}\right)}^{2}}{N}}$$

## Methods

In this research application of all the five machine learning techniques for the case of Rwanda have been applied, to predict the out-of-pocket health expenditures in Rwanda using EICV5 dataset collected from national institute of Rwanda, a sample of 14580 households was used and techniques were: decision tree models, treenet models, random forest model, MARS model, gradient boosting, R programming and Alteryx have been used as tools.

**Data:** the current study uses secondary data analysis from integrated living conditions survey in Rwanda (EICV5) 2016-2017 conducted by national institute of statistics of Rwanda to gather up-date information for out-of-pocket health expenditures at household level in Rwanda. In this study, out-of-pocket health expenditures, total consumption, ownership of assets and consumption at household level, were simultaneously considered. It dealt with quantitative research approach as numerical information were collected.

**Study setting:** the study was conducted in Rwanda, with 30 districts with estimated total population of 11.8 million. Furthermore, 48% of men and 52% of women, distributed in five provinces and 30 districts. Rwanda is surrounded by four countries such as Uganda in the North, Tanzania in the East, Burundi in the South and Republic of Congo in the West and 35.1% of the population is urban and the rest 64.9% live in rural areas [24].

**Source of data:** this secondary data was collected from national institute of statistics of Rwanda for the integrated living conditions survey 2016-1017 (EICV5), mainly focusing on variables includes: age, sex, households´ size, household expenditure, region, household insurance status, out-of-pocket health expenditure, total consumption, ownership of assets.

**Target population:** the data used for this analysis is from integral living conditions survey 2016-2017 (EICV5) conducted by the national institute of statistics of Rwanda. This nationally representative survey gathered data from 14580 households and 64314 individuals. Information was collected at the household and the individual level [24]. Household level information included consumption expenditure on food, non-food items and out-of-pocket health expenditures including: consultation; laboratory tests; hospitalization; and medication costs. Individual level information included socio-economic indicators and insurance status, self-reported health need and utilization of health services. However, there was a recall period for utilization of health services in two weeks. Different recall period from the survey were used to improve reliability of OOP data. These were two weeks for outpatient services expenditure and twelve months for inpatient services expenditure. In this research application of all the five machine learning techniques for the case of Rwanda has been applied, those techniques were: decision tree models, gradient boosting models, treenet model, random forest model, MARS model.

**Dependent variable:** dependent of the study is the out-of-pocket health which depends on 14 variables. Those variables play vital role in predicting the out-of-pocket health expenditure in Rwanda.

**Independent variables:** for this study, 14 independent variables were hypothesized to influence the dependent variable. These variables include household size, total consumption, age of the head of the household, provinces, poverty rate, households owning at least one car, households owning at least one motorcycle, households owning at least one refrigerator, households owning at least one cooker machine, households owning at least one improved sanitation, households owning at least one bicycle, households owning at least toilet not shared, households owning at least one improved sanitation, sex of the head of the household, that may affect the dependent variable. Finally, selection of independent variables is based on the past research and published literature related to the study.

## Results

The aim of these empirical results was to develop a model for estimating out-of-pocket health expenditure in Rwanda at household level using machine learning techniques.

**Figure 1:**
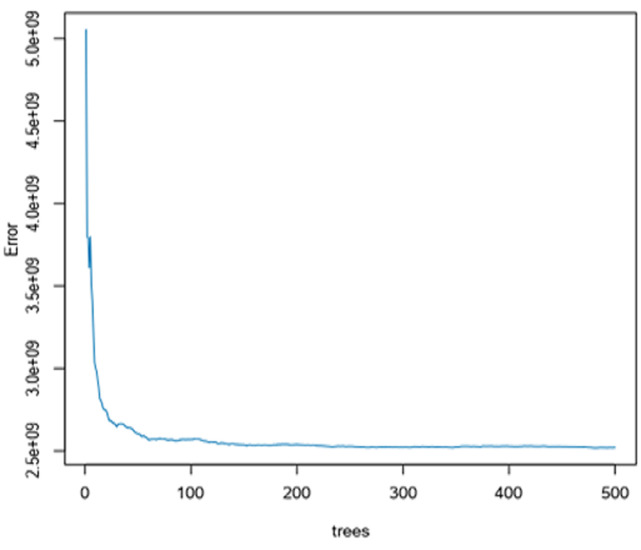
random forest model

**Figure 2:**
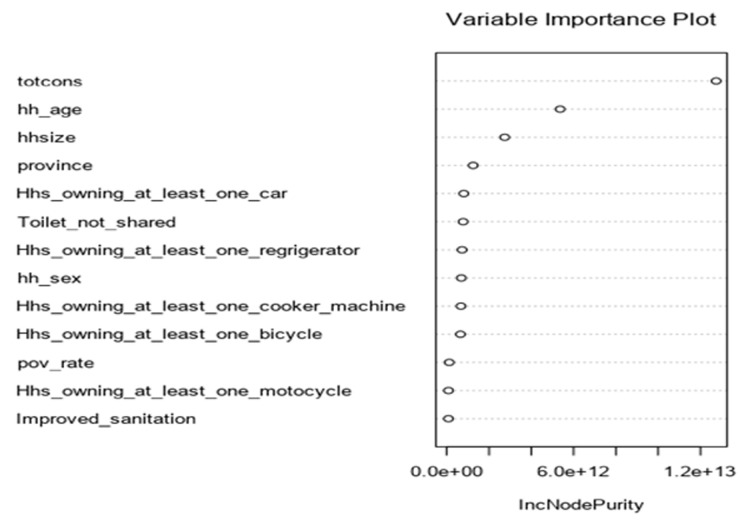
variable importance for random forest model

**Random forest was developed:** the percentage error in random forest model reduced as long as we increase the numbers of tree at the range of more than 500 trees (Figure 1) and for variable importance the total consumption contributes a lot to the random forest model than other predictors and the total consumption played a vital role as the most important variable in random forest model but it is also followed the age of the head of household and household size respectively and other variables were neglected. However, the errors may reduce in case we increase the number of trees, may be more than 500 trees. Finally, the percentage of variance explained is 83% (Figure 2).

**Figure 3:**
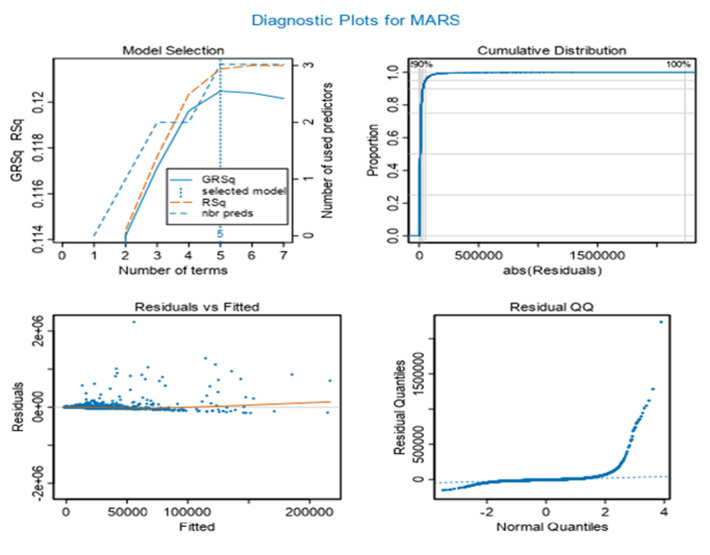
MARS model

**MARS model was developed:** the four plots in the set of diagnostic plots are: the model selection plot; the cumulative distribution plot; residuals vs fitted plot; and the normal Q-Q plot. The model selection plot gives the RSq (R-squared) and GRsq (penalized R-squared of the model), while the dashed line gives the number of terms in the model. The cumulative distribution plot shows the cumulative distribution of the absolute values of the model residuals, which ideally starts at zero and quickly rises to one (Figure 3).

The residuals vs fitted value plot shows the residual for each value of the predicted response. By comparing the scales of the two axes, one can quickly gain a sense of the size of the residuals relative to the predictive values. The thin line in the plot indicates how the average magnitude of the residual´s changes with the size of the predictive value. Ideally, this would be essentially a horizontal line centered at zero of the residuals (vertical) axis of the plot (Figure 3).

**Figure 4:**
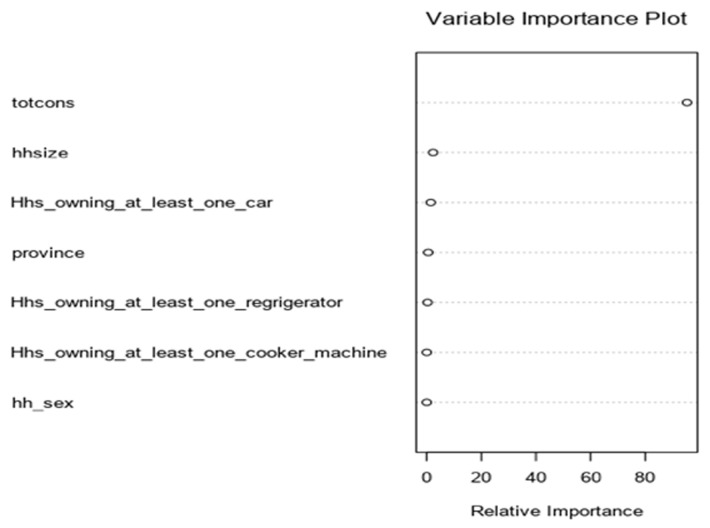
variable importance for MARS model

The normal Q-Q plot compares the distribution of the residual to a normal distribution. Deviations for normality are only critical when the target is continuous and the Gaussian GLM family is selected. Its value for other models is the ability to see potential outliers in the data. Finally, the model with the highest GRSq value is selected and the vertical dotted line indicates the number of model terms included in this best model and variable importance for MARS. The variable importance plot provides information about the relative importance of each predictor field. The measures are normalized to sum to 100 and the value for each field gives the relative percentage importance of that field to the overall model, for total consumption played a significant role on MARS model up to 78%. Finally, three features among 14 predictors played a significant role for MARS model were total consumption, age of the head of household and household size (Figure 4).

**Figure 5:**
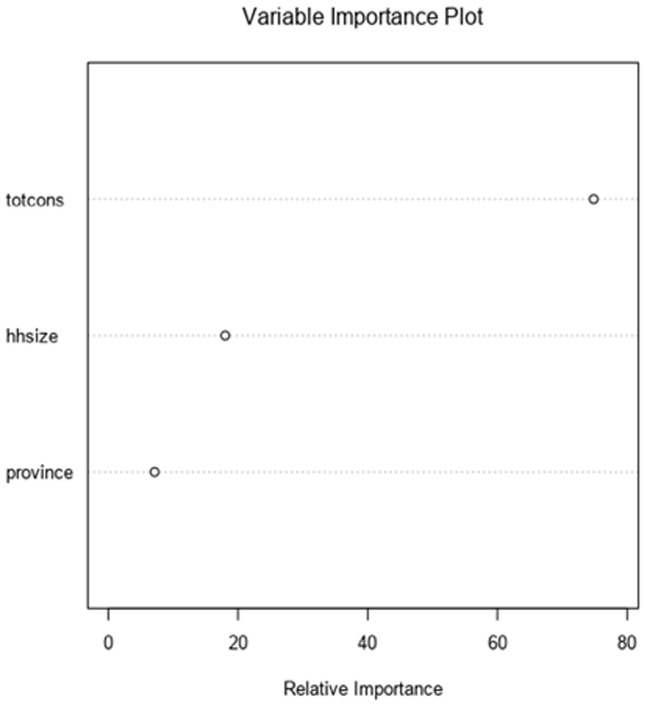
variable importance for treenet model

**Figure 6:**
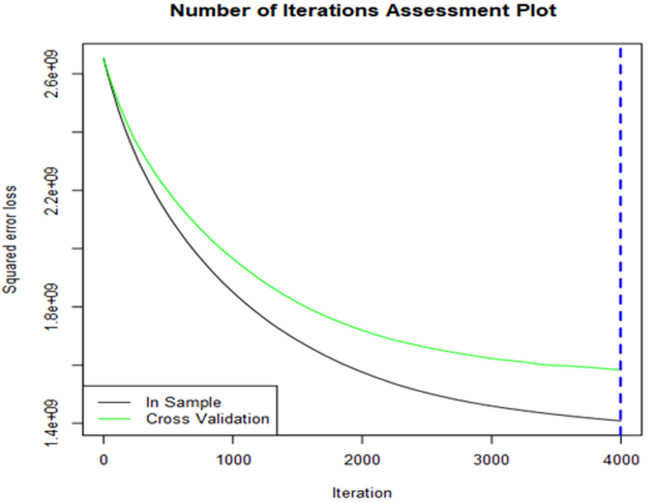
cross-validation for treenet model

**Treenet model was developed:** the variable importance plot provides information about the relative importance of each predictor field. The measures are normalized to sum to 100 and the value for each field gives the relative percentage importance of that field to the overall model and consumption has high impact of the model than other variables for 85% (Figure 5). However, the number of iterations assessment plot illustrates how the deviance (loss) changes with the number of trees included in the model. Finally, the vertical blue dashed line indicates where the minimum deviance occurs using the specified assessment criteria (cross validation, the use of a test sample or out-of-bag prediction) (Figure 6).

For the coefficients for MARS model the three variables were selected among other variable to describe the hinge function of MARS model and this indicate how these three variables influence the out-of-pocket health expenditures in positive way or negative way by considering non linearity between variables, thus three features among 14 predictors played a significant role for MARS model were total consumption, age of the head of household and household size (Table 1).

**Table 1:** MARS model coefficients with non-linearity between variables used in this analysis

Term	Value
Intercept	1.164e+05
H (totcons-1.09e+07)	2.021e-03
H (1.09e+07-totcons)	-9.936e-03
H (4-hhsize)	-3.568e+03
H (province-3)	6.165e+03

**Root mean square error measures were calculated to select which model was the best to predict the out-of-pocket health expenditures in Rwanda:** for the test data, the result for root mean square error metrics was 2188 for treenet model and R^2^ was 87%. Finally, we can confirm that the treenet model perform well than decision tree model and gradient boosting model. As the cost of misclassification for out of sample with machine learning feature selection is considered, the tests of accuracy of the model are 50.16% for MARS model, 74% decision tree model, 87.3% for treenet model, 83% for random forest, gradient boosting 81%.

Table 2 indicates that the present yearly average amount spent by household was 20185Rwf and each machine learning model predict the out-of-pocket health expenditures, for that reason treenet model predict 21457Rwf, random forest 22421Rwf, gradient boosting 25165Rwf, decision tree 22870Rwf, MARS model 21990Rwf (Table 3).

**Table 2:** root mean square measures indicate the yearly average of amount spent by household in the out-of-pocket health expenditures in Rwanda with machine learning models

ID	Model	MAE	MSE	RMSE	R_squared
1	Treenet model	927.599943	2.287597e+07	2188.4	0.87
2	Rforest	469.074179	4.789224e+06	4782.8	0.83
3	Gboost	2087.421770	1.545435e+07	3931.2	0.81
4	Decision tree	3829.66	3.23556e+6	3124	0.74
5	MARS model	2154.12	1.3455e+5	2467	50.16%

**Table 3:** prediction results of out-of-pocket health expenditures in Rwanda with machine learning models

ID	Machine learning models model	Yearly average out of pocket health expenditures in Rwanda	Predicted yearly average out of pocket health expenditures in Rwanda
1	Treenet model	20185Rwf	21457Rwf
2	Random forest	20185Rwf	22421Rwf
3	Gradient boosting	20185Rwf	25165Rwf
4	Decision tree	20185Rwf	22870Rwf
5	MARS model	20185Rwf	21990Rwf

## Discussion

The present study examined the best model which performs well in predicting the out-of-pocket health expenditures in Rwanda. Furthermore, we assessed how out-of-pocket health expenditures can be predicted using machine learning models. The results from Table 2 indicate that treenet model perform well than other considered models in predicting this out-of-pocket health expenditures based on its root mean square error and coefficient of determination and these calculated for training data set and for test data set. Higher coefficient of correlation for both training and test dataset and lower root mean square was considered as the best one. Referring to the Table 2 the yearly average out-of-pocket health expenditures in Rwanda was 20185Rwf per household.

Apart from root mean square results, the findings from Table 3 showed that the predicted out-of-pocket health expenditures in Rwanda, the selection of the best model was indicated by the smallest predicted amount which was treenet model with 21457Rwf expected amount spent at household level in health expenditures. Figure 1 indicates that the random forest model produces the errors up to the 500 trees, this means that to reduce the error in the random forest model we need to increase the number of trees up to more than 500 trees.

Figure 2 indicates variable importance in the random forest model is the consumption, it means that the consumption has high impact on random forest model. Figure 3 indicates the selection of model is based to the residual root mean square indicated by dots in red color. Figure 4 indicates variable importance for MARS model is the consumption, Figure 5 indicates variable importance for treenet model is also consumption, Figure 6 indicates cross-validation for treenet model in sample and in cross-validation data set indicated in the blue curve.

Reference made to the findings from Thomson *et al*. (2018), our findings showed that the treenet model had the best predictive performance to predict the out-of-pocket health expenditures in Rwanda.

## Conclusion

The purpose of this research paper has been achieved through the results we got in predicting the out-of-pocket health expenditures in Rwanda. However, compared to traditional statistics used in predicting the out-of-pocket health expenditures that often criticized due to their model assumptions. Furthermore, machine learning approach is becoming a real alternative in predicting the out-of-pocket health expenditures due to its internal memory characteristic, generalization capability. This paper has investigated the training and test accuracies of the models using EICV5 dataset from national institute of statistics of Rwanda. The findings of this paper suggest that in training and test among machine learning model models, treenet model was much better in terms of accuracy. Even though, the conclusions in this paper need to be validated with other data sets in future researches, a general conclusion can be drawn from this study: machine learning feature selection technique provides an effective method for selecting the most predictable features and machine learning scoring models. Finally, we conclude that the total consumption of the household came out to be the most important variable which is consistently true to all the algorithms tested. The findings from our study have policy implications for policy makers in Rwanda and in the world generally. Finally, governments have to significantly increase public spending on health. Domestic financial resources are key to moving closer to universal health coverage (UHC) and should be increased on a long-term basis.

### What is known about this topic

- Health problems are causing not only suffering and death but also negatively affect financial sides;
- For health insurers and increasingly healthcare delivery systems, accurate prediction of likely costs can help with general business planning.

### What this study adds

- To provide the best model which can predict with accuracy the out-of-pocket health expenditures in Rwanda;
- The yearly average present and future amount spent at household level in health expenditures;
- For patients, knowing in advance their likely health expenditures for the following year could potentially allow them to choose insurance plans with appropriate deductibles and premiums.

## References

[1] Jones AM (2010). Models for health care. HEDG Department of Economics, University of York.

[2] Marshall AH, Shaw B, McClean SI (2007). Estimating the costs for a group of geriatric patients using the Coxian phase-type distribution. Stat Med.

[3] Breiman L (2001). Random forests. Machine Learning 45, fourth edition.

[4] Mihaylova B, Briggs A, O'Hagan A, Thompson SG (2011). Review of statistical methods for analyzing healthcare resources and costs. Health Econ.

[5] Patriche CV, Pirnau RG, Rosca B (2011). Comparing linear regression and regression trees for spatial modelling of soil reaction in basin (eastern romania). Bulletin UASVM Agriculture.

[6] Lahiri B, Agarwal N (2014). Predicting healthcare expenditure increase for an individual from medicare data. In Proceedings of the ACM SIGKDD Workshop on Health Informatics.

[7] Cylus J, Thomson S, Evetovits T (2018). Catastrophic health spending in Europe: equity and policy implications of different calculation methods. Bull World Health Organ.

[8] Bertsimas D, Bjarnadóttir MV, KaneMA Kryder JC, Pandey R, Vempala S (2008). Algorithmic prediction of health-care costs. Operations Research.

[9] Friedman JH (2001). Greedy function approximation: a gradient boosting machine. Ann Stat.

[10] Getzen T (2000). Forecasting health expenditures: short, medium and long (long) term. J Health Care Finance.

[11] McWilliams JM, Meara E, Zaslavsky AM, Ayanian JZ (2007). Use of health services by previously uninsured Medicare beneficiaries. N Engl J Med.

[12] Mitchell TM (1997). Machine Learning.

[13] Ortiz I, Schmitt V, De L (2016). Social protection floors volume 1: universal schemes. International Labour Organization (ILO).

[14] Kawabata K, Xu K, Carrin G (2002). Preventing impoverishment through protection against catastrophic health expenditure. Bull World Health Organ.

[15] Kronick R, Gilmer TP, Dreyfus T, Ganiats TG (2002). CDPS-Medicare: the chronic illness and disability payment system modied to predict expenditures for medicare beneciaries.

[16] Rokach L, Maimon O (2008). Data mining with decision trees: theory and applications.

[17] Pope GC, Kautter J, Ellis RP, Ash AS, Ayanian JZ, Lezzoni LI (2004). Risk adjustment of medicare capitation payments using the cms hcc model. Health Care Financ Rev.

[18] Lee T-S, Chiu C-C, Lu C-J (2006). Mining the customer credit using classification and regression tree and multivariate adaptive regression splines. Computational Statistics & Data Analysis.

[19] Thomson S, Evetovits T, Cylus J (2018). Financial protection in high income countries. A comparison of the Czech Republic Estonia and Latvia.

[20] World Health Organization (2019). Can people afford to pay for health care. new evidence on financial protection in Europe.

[21] Huber CA, Schneeweiss S, Signorell A, Reich O (2013). Improved prediction of medical expenditures and health care utilization using an updated chronic disease score and claims data. J Clin Epidemiol.

[22] Sophie Faye (2013). The economics of health care in Senegal: a dissertation submitted to the faculty of the college of arts and sciences in partial fulfilment of the degree requirements for the doctor of philosophy department of economics.

[23] Manning WG, Basu A, Mullahy J (2005). Generalized modeling approaches to risk adjustment of skewed outcomes data. J Health Econ.

[24] Wang W, Temsah G, Carter E (2016). Levels and determinants of out-of-pocket health expenditures in the Democratic Republic of the Congo, Liberia, Namibia and Rwanda. ICF International.

